# T-cells engineered with a novel VHH-based chimeric antigen receptor against CD19 exhibit comparable tumoricidal efficacy to their FMC63-based counterparts

**DOI:** 10.3389/fimmu.2023.1063838

**Published:** 2023-02-16

**Authors:** Fatemeh Nasiri, Pooria Safarzadeh Kozani, Fatemeh Rahbarizadeh

**Affiliations:** ^1^ Department of Medical Biotechnology, Faculty of Medical Sciences, Tarbiat Modares University, Tehran, Iran; ^2^ Research and Development Center of Biotechnology, Tarbiat Modares University, Tehran, Iran

**Keywords:** chimeric antigen receptor, CD19, cancer immunotherapy, hematologic malignancy, VHH, scFv

## Abstract

**Background:**

Chimeric antigen receptor (CAR)-T cell therapy has established itself as a potent therapeutic option for certain patients with relapsed/refractory (R/R) hematologic malignancies. To date, four CD19-redirected CAR-T cell products have been granted the United States Food and Drug Administration (FDA) approval for medical use. However, all of these products are equipped with a single-chain fragment variable (scFv) as their targeting domains. Camelid single-domain antibodies (VHH or nanobody) can also be used as alternatives to scFvs. In this study, we developed VHH-based CD19-redirected CAR-Ts, and compared them with their FMC63 scFv-based counterpart.

**Methods:**

Human primary T cells were transduced to express a second-generation 4-1BB-CD3ζ-based CAR construct whose targeting domain was based on a CD19-specific VHH. The expansion rate, cytotoxicity, and secretion of proinflammatory cytokines (IFN-γ, IL-2, and TNF-α) of the developed CAR-Ts were assessed and compared with their FMC63 scFv-based counterpart as they were co-cultured with CD19-positive (Raji and Ramos) and CD19-negative (K562) cell lines.

**Results:**

VHH-CAR-Ts showed an expansion rate comparable to that of the scFv-CAR-Ts. In terms of cytotoxicity, VHH-CAR-Ts mediated cytolytic reactions against CD19-positive cell lines, comparable to those of their scFv-based counterparts. Moreover, both VHH-CAR-Ts and scFv-CAR-Ts secreted remarkably higher and similar levels of IFN-γ, IL-2, and TNF-α upon co-cultivation with Ramos and Raji cell lines compared with while cultured alone or co-cultured with K562 cells.

**Conclusion:**

Our results demonstrated that our VHH-CAR-Ts could mediate CD19-dependent tumoricidal reactions as potently as their scFv-based counterparts. Moreover, VHHs could be applied as the targeting domains of CAR constructs to overcome the issues associated with the use of scFvs in CAR-T therapies.

## 1 Introduction

Cancer immunotherapy has changed the face of the fight against cancer in the past decades. The advent of different platforms of cancer immunotherapy has proven its effectiveness for the treatment of a wide range of immunological and oncological indications (1, 2). Monoclonal antibodies (mAbs), T-cell-redirecting bispecific antibodies (TRBAs), antibody-drug conjugates (ADCs), tumor-infiltrating lymphocytes (TILs), and chimeric antigen receptor T cells (CAR-Ts) are now known as revolutionary second-, third-, fourth-, and fifth-line treatments for special groups of patients. CAR-T therapy has experienced a rapid progress in the past decade as the US Food and Drug Administration (FDA) approved six products for five different malignancies (3–15). So far, CAR-Ts have been granted approval for the treatment of patients with hematologic malignancies which include B-cell precursor acute lymphoblastic leukemia (B-ALL; *tisagenlecleucel* and *brexucabtagene autoleucel*), diffuse large B-cell lymphoma (DLBCL; *tisagenlecleucel*, *axicabtagene ciloleucel*, and *lisocabtagene maraleucel*), follicular lymphoma (FL; *tisagenlecleucel* and *axicabtagene ciloleucel*), mantle cell lymphoma (MCL; *brexucabtagene autoleucel*), and multiple myeloma (MM; *ciltacabtagene autoleucel* and *idecabtagene vicleucel*) (16). It is interesting to mention that four of the approved CAR-T products target CD19 as their target antigen while the other two (namely, *ciltacabtagene autoleucel* and *idecabtagene vicleucel*) target B-cell maturation antigen (BCMA).

CD19 has been an interesting target antigen for immunotherapy (17, 18). To date, aside from four FDA-approved CD19-redirected CAR-T products, one TRBA (*Blinatumomab*), two humanized mAbs (*Tafasitamab* and *Inebilizumab*), and one humanized ADC (*Loncastuximab tesirine*) have also been granted FDA approval against CD19, which accentuates its potential as one of the most, if not the most, successful target antigens of cancer immunotherapy. Also, numerous clinical trials are currently investigating the safety and efficacy of different CD19-redirected CAR T cells in Phase II and III (NCT05281809, NCT04605666, NCT04257175, etc.).

The production of a conventional CAR-T product entails blood sampling from the patient, isolation of peripheral blood mononuclear cells (PBMCs), genetic engineering of the isolated T cells for the expression of CAR molecules, and reinfusion of the generated CAR-Ts into the patient (19). A CAR molecule is topologically made from three domains; the extracellular domain, the transmembrane domain, and the intracellular domain. The intracellular domain of CARs is where the signaling domains are incorporated (4-1BB-CD3ζ or CD28-CD3ζ in the case of the FDA-approved CAR-Ts) while the extracellular domain redirects the cytotoxicity of CAR-Ts against cells expressing the target antigen (for which the targeting domain of CAR is specific) (16). CAR targeting domains are usually derived from the single-chain fragment variable (scFv) of mAbs; however, researchers have recently applied other targeting domains such as single variable domain on a heavy chain (VHH; also referred to as nanobodies), peptides, and ligands (20). Of note, the targeting domain of Janssen’s *ciltacabtagene autoleucel* is based on BCMA-specific single-domain antibodies (20). Such efforts alongside successful clinical records prove that CAR-T products could be developed using targeting domains other than scFvs (20). In this research, we constructed a VHH-based CD19-redirected CAR molecule using a CD19-specific VHH previously isolated in our laboratory, and developed VHH-based CD19-redirected CAR-Ts (hereafter referred to as VHH-CAR-Ts) (21). Then, we assessed the tumoricidal efficacy of the VHH-CAR-Ts against CD19-positive cell lines to demonstrate that they can be as potent as FMC63 scFv-based CAR-Ts *in vitro*.

## 2 Materials and methods

### 2.1 Cells

All of the cell lines used in this study were purchased from National Cell Bank of Iran (NCBI), and Pasteur Institute (Tehran, Iran), and were cultured in a humidified condition at 37°C (5% CO_2_). Human embryonic kidney 293T (HEK293T) cells were cultured in high glucose Dulbecco’s Modified Eagles Medium (DMEM; Gibco, Life Technologies, USA) supplemented with 10% (v/v) fetal bovine serum (FBS; Gibco, Life Technologies, USA), 1 mM sodium pyruvate, 4 mM L-glutamine, and 1% penicillin-streptomycin (100 IU/mL). HEK293T cells were used for the production of lentiviral particles and the titration of the produced lentiviruses. Burkitt’s lymphoma cell lines Raji and Ramos were used as CD19-positive human blood cancer cells, and K562 was used as CD19-negative human blood cancer cells, and were cultured and maintained in Roswell Park Memorial Institute (RPMI) 1640 (Gibco, Life Technologies, USA) supplemented with 4 mM L-glutamine, 1 mM sodium pyruvate, 1% penicillin-streptomycin (100 IU/ml), and 10% (v/v) FBS. Raji, Ramos, and K562 cells were applied for evaluating the specific cytotoxicity, proliferation, and cytokine secretion of VHH-CAR-Ts and scFv-CAR-Ts upon co-cultivation.

Human blood samples were collected from healthy donors (HD; n = 3) after obtaining written informed consent according to the guidelines and the approval of *Tarbiat Modares University Research Ethics Committee*. PBMCs were isolated using density gradient centrifugation by diluting the obtained blood in phosphate-buffered saline (PBS; with a ratio of 1:1 v/v) and slowly layering it over Ficoll-Hypaque (Lymphodex, Inno-Train, Germany) followed by centrifugation at 800 × g for 20 minutes at room temperature. Then, the PBMC layer was harvested carefully and washed twice with PBS. Ultimately, the obtained primary T cells were cultured in complete RPMI 1640 media (10% FBS v/v) supplemented with 50 IU/mL interleukin (IL)-2 (MACS, Miltenyi Biotec, BIOTEC GmbH, Germany). Dynabeads™ Human T-Activator CD3/CD28 (Gibco, Thermo Fisher Scientific, USA; catalog No. #11131D) were used to activate primary T cells at a bead-to-cell ratio of 2:1. Lentiviral transduction with the desired multiplicity of infection (MOI) was carried out on the activated T cells after three days of incubation at 37°C (5% CO_2_).

### 2.2 Plasmids and reagents

The pLJM1 lentiviral vector encoding the green fluorescent protein (*PLJM1-EGFP*; Cat. No. #19319; Addgene, MA, USA) was used as the GFP control transfer vector (lentiviral control used for the transduction of T cells without CAR expression, hereinafter referred to as CAR-negative cells) in this study for virus production, titration steps, and functional assays. Two other versions of this vector were also generated; one encoding “anti-CD19.VHH.CAR-4-1BB-CD3ζ” as our main transfer vector (called *pLJM1VHH19*), and the other one encoding “anti-CD19.scFv.CAR-4-1BB-CD3ζ” (called *pLJM1scFv19*) applied as the positive control. Of note, pLJM1scFv19 harbors the same CAR construct as pLJM1VHH19 with the only exception that the targeting domain of pLJM1scFv19 is composed of the CD19-specific scFv, FMC63, rather than our CD19-specific VHH. To transfer the CAR transgenes into T cells, third-generation lentiviruses were used. In this regard, two packaging plasmids, “*pMDLg/pRRE*” and “*pRSV/Rev*”, and one envelope plasmid, “*pMD2G*”, were used alongside the transfer vector in the co-transfection process for the encapsulation of the third-generation lentiviruses. Moreover, polyethylenimine (PEI; 25 kDa; Cat. No. #9002-98-6, Sigma-Aldrich, Merck KGaA, Germany) was used as the transfection enhancer/reagent during the process of plasmid co-transfection and lentiviral packaging.

### 2.3 CAR construct

The VHH-based CD19-redirected CAR cassette contained the coding sequences for a CD19-specific VHH along with CD8α, followed by the 4-1BB costimulatory domain and the CD3ζ signaling domain, all of which were synthesized by Genscript (Piscataway, NJ, USA). This construct was PCR-amplified using the following oligonucleotide primers 5’ GCTAGCATGGCCTTACCAGTG 3’ and 5’ TTCGAACTAGCGAGGGGGCAG 3’ as forward and reverse primers, respectively. The forward and reverse primers were designed to introduce NheI (New England Biolabs, MA, USA) and BstbI (New England Biolabs, MA, USA) restriction sites at the 5’ and 3’ end of the CAR construct, respectively. The resultant CAR construct was subcloned into the pLJM1-EGFP vector using the mentioned restriction enzymes and T4 DNA ligase (Thermo Fisher Scientific, MA, USA). Finally, plasmid extraction was carried out using the Geno pure Plasmid Maxi Kit (Roche; Cat No. #03143422001) as an endotoxin-free kit for large-scale plasmid DNA isolation with sufficient high-quality.

### 2.4 Co-transfection and lentiviral packaging

12 hours prior to co-transfection, HEK293T cells (1.5 × 10^6^; at a confluency rate of ~80%) were seeded in 6-well treated tissue culture plates. 10 hours later, the media was replaced with fresh media containing 2% v/v FBS. Next, the polyplex supernatant for lentiviral production was prepared by co-transfecting a lentiviral transfer vector (PLJM1-EGFP, pLJM1VHH19 or pLJM1scFV19), and two lentiviral packaging vectors and one envelope vector at a ratio of 4:(2:2):1, respectively, using PEI as the transfection reagent. Four different ratios of PEI:DNA (w/w; 1:1, 2:1, 3:1, and 4:1) were tested during co-transfection to determine the PEI:DNA mixture ratio with lowest toxic effects yielding the maximum viral titration. The GFP-expressing cells were detected under a fluorescence microscope, 24 to 72 hours after transfection. Furthermore, the transfection efficiency for the optimal non-toxic ratio of PEI:DNA was determined using flow cytometry analysis (BD FACSCalibur flow cytometer, BD Biosciences, San Jose, California, USA) 24 hours after transfection. For this aim, the cells were stained with propidium iodide (PI; Sigma-Aldrich, Merck KGaA, Germany) before flow cytometry analyses. For data analysis, the population of cells were first gated on PI-negative cells (live cells) and then the expression of GFP was assessed in that population. Also, the titration of the produced lentiviral particles was assessed using flow cytometry analysis and quantitative PCR (qPCR) 72 hours after transduction in every sample. Of note, the virus-containing supernatant was collected 24, 48, and 72 hours after transfection, concentrated using ultracentrifugation, and stored at -80°C for the further steps of the study.

### 2.5 Genome integration analysis for determining viral titration

To analyze the quality and quantity of the recombinant viral particles, the flow cytometry assay was used for the assessment of GFP and CAR expression in the relative groups, and quantitative PCR (qPCR) to determine vector copy numbers. For this aim, HEK293T cells (2 × 10^4^ per well) were seeded into a treated 96-well tissue culture plate with incomplete DMEM media overnight prior to transduction. In the following step, the supernatant samples containing lentiviral particles were thawed on ice, and multiple serial dilutions of them were prepared (10^−1^, 30^−1^,10^−2^, 30^−2^, 10^−3^, and 30^−3^) in DMEM high glucose media containing 8 μg/mL polybrene (hexadimethrine bromide, Sigma-Aldrich, Merck KGaA, Germany), and then they were incubated 30 minutes at 37°C (5% CO_2_). Seeded HEK293T cells were transduced with lentivirus serial dilutions and were incubated for 12 hours at 37°C (5% CO_2_), followed by an exchange of fresh complete DMEM media, and incubation. For the flow cytometry assay, the transduced cells were trypsinized 72 hours after transduction (to ensure gene expression), then the cells were suspended in PBS. Finally, the expression of the transgene of each group (GFP, scFv-based CARs, and VHH-based CARs) was evaluated by flow cytometry for determining viral titers in transduction units (TU)/mL. Moreover, the genomic DNA was extracted from the transduced HEK293T cells using a High Pure PCR Template Purification Kit (Roche, Mannheim, Germany) for calculating the copy number of the integrated transgenes using a concentration standard curve (with known titers). To verify that free plasmid was removed from the media, q-PCR was performed after two passages (every three days over the course of 10 days) with primers specific for the puromycin gene (the viral resistance gene as an integrated sequence). qPCR was conducted on a Corbett Rotor-Gene 6000 instrument (Corbett Life Science; Sydney, Australia) using the following materials: 10 μL of Taq DNA Polymerase 2x Master Mix RED (Ampliqon; Cat. No. #A190303), 0.4 μM of each primer, template and distilled nuclease-free water to reach the final volume of 20 μl. The qPCR was performed in two steps with the following thermal settings: 15 minutes at 95°C for an initial Taq DNA polymerase activation step with hot start followed by 40 amplification cycles (30 seconds at 95°C and 30 seconds at 60°C). The test samples, standards, and negative and positive controls were all performed in duplicate. The cycle threshold (Ct) was set at 37 ± 2 since it does not adversely affect detection sensitivity. Of note, the forward and reverse puromycin primers were 5’ GCAGCAACAGATGGAAGG 3’ and 5’ GAGGTCTCCAGGAAGGC 3’, respectively.

### 2.6 T cell lentiviral transduction

Primary T cells were activated using Dynabeads™ Human T-Activator CD3/CD28 and 100 IU/mL IL-2 while being incubated for 3 days prior to lentiviral transduction. Next, the media of the activated cells were replaced with concentrated lentiviral supernatant supplemented with polybrene at a concentration of 6 μg/mL of culture media. Next, the cells were centrifuged for 60 minutes at 1200 × g at 25°C. The stimulation beads were removed after 6-8 days of culture following transduction. Also, T cell culture media was supplemented with 50 IU/mL IL-2 three times per week. For flow cytometry analysis, the population of the cells were first gated on CD3-positive cells, and then the percentage of CAR-positive cells was analyzed in each cell population.

### 2.7 Target antigen-triggered activation and proliferation of CAR-Ts

The target cell-triggered expansion capability of the CAR-Ts was analyzed using the carboxyfluorescein succinimidyl ester (CFSE; BioLegend, San Diego, CA; Cat No. 423801) cell staining assay (22). Briefly, 10^6^ - 10^7^ effector T cells were labeled with 1 μL of 5 mM CFSE per mL of PBS. After a 20-minute incubation in a dark condition at 37°C (5% CO_2_), the stained cells were resuspended in PBS, washed twice, and incubated overnight in culture media. The CFSE-stained effector cells were co-cultured with stimulator target cell lines without IL-2 at an effector-to-target (E:T) ratio of 6:1. After 72 hours of incubation, the effector cells were stained with APC-conjugated anti-human CD3 antibody and the CFSE signal reduction through the CD3+ cells division was assessed by flow cytometry. Also, the Mitomycin C inactivation protocol was used for inhibiting the proliferation of target cells. Briefly, 10^6^ target cells were incubated with 50 μL of 1 mg/mL mitomycin C for 1 hour before co-culturing with the respective effector cells.

### 2.8 Cytotoxicity assessment

A cytotoxicity test was conducted by staining the target cells of CAR-Ts with PI while gating on CFSE-labeled target cells. In detail, CAR-Ts were co-cultured with CFSE-stained (in accordance with the previous protocol) K562, Raji, and Ramos target cells at different E:T ratios (3:1, 6:1, and 10:1) for 16 hours followed by PI staining to measure cell death *via* flow cytometry. Target cells included the CD19-negative cell line K562 (used as negative control) and the CD19-positive cell lines Raji and Ramos. For flow cytometry analysis, the population of cells was first gated on CFSE-positive cells, and then the percentage of apoptotic cells was assessed in that population (as PI-positive cells).

### 2.9 Inflammatory cytokine production of CAR-Ts

The level of CAR-T-secreted cytokines including human IFN-γ (human IFN-γ ELISA Kit; ab46025, Abcam, MA, USA), human TNF-α (human TNF-α ELISA Kit; ab46087; Abcam, MA, USA), and human IL-2 (human IL-2 ELISA Kit; ab100566; Abcam, MA, USA) was measured in the supernatant of the cells *via* enzyme-linked immunosorbent assay (ELISA) according to the manufacturer’s instructions. Briefly, CAR-Ts were co-cultured with the target cells at an E:T ratio of 6:1, and the supernatant was collected 24 hours after incubation.

### 2.10 Flow cytometry antibodies and antigens

FITC-conjugated human CD19 antigens (Cat. No. CD9-HF2H2; Acro Biosystems, Newark, NJ, USA) were used for determining the expression rate of the VHH-based and scFv-based CAR molecules on the surface of VHH-CAR-Ts and scFv-CAR-Ts, respectively, in the flow cytometry assay. The concentration of the FITC-conjugated CD19 antigen and the number of cells used for the flow cytometry assay were according to the manufacturer’s instructions. APC-conjugated anti-human CD3 antibody (BD Pharmingen™, USA; Cat No. #555335) was used to assess the percentage of CD3-positive cells in a given population of PBMCs, according to the manufacturer’s instructions.

### 2.11 Statistical analyses

Statistical analyses were performed using GraphPad Prism version 8.01 (Graph Pad Software, Inc., USA). The mean values for each group were calculated, and the error bars indicate the standard deviation. Statistical significance was determined using one-way ANOVA with Tukey’s multiple comparison test. Significances are represented as * for p values < 0.05, ** for p values < 0.01, *** for p values < 0.001, and **** for p values < 0.0001.

## 3 Results

### 3.1 PEI:DNA ratio optimization and lentiviral packaging

**Figure 1:**
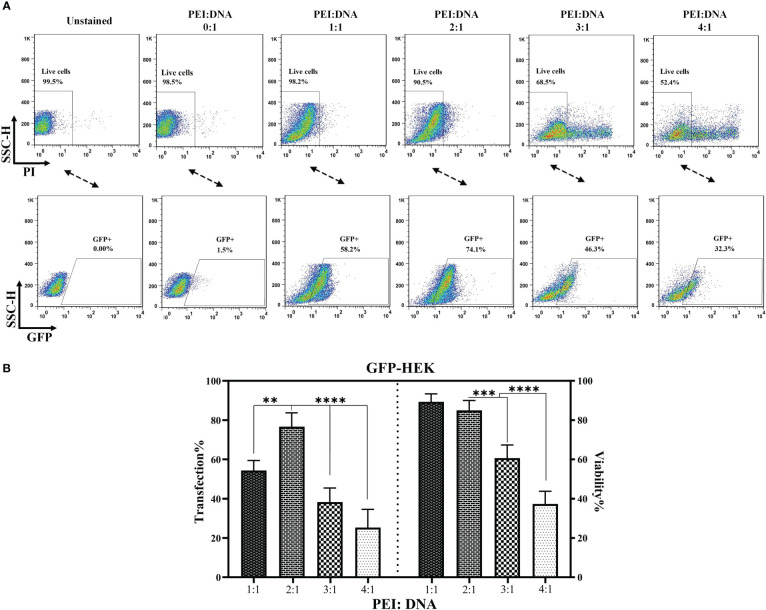
PEI:DNA ratio optimization for lentiviral packaging. **(A)** Flow cytometry plots for GFP-expressing cells and the post-transfection viability of HEK293T cells. **(B)** Statistical analysis between different groups of HEK293T cells transfected using four different PEI:DNA ratios. Data are expressed as the mean ± SD. **p < 0.01, ***p < 0.001, and ****p < 0.0001. All experiments were carried out in triplicate.

The percentage of living cells as well as the percentage of cells expressing GFP were quantified 24 hours after transfection to determine the most appropriate PEI:DNA ratio. As presented in **Figures 1A, B**, PEI:DNA ratios of 1:1 and 2:1 did not result in a remarkable decline in the level of living HEK293T cells; however, the 3:1 and 4:1 ratios mediated significant cytotoxicity against these cells, resulting in 68.5 and 52.4% living cells, respectively. In terms of GFP expression, the highest proportion of GFP-expressing cells was detected in the 2:1 and 1:1 PEI:DNA ratio groups with 74.1 and 58.2%, respectively. Overall, the PEI:DNA ratio of 2:1 resulted in the highest percentage of GFP-expressing HEK293T cells and the lowest rate of cells damaged by the cytotoxic effects of PEI; therefore, this ratio was selected as the optimized PEI:DNA ratio. Also, according to the statistical analysis (presented in **Figure 1B**), the PEI:DNA ratio of 2:1 mediated a significantly higher rate of transfection in comparison with other PEI:DNA ratios (p value < 0. 01 for PEI:DNA ratio of 1:1 and 2:1, and p value < 0.0001 for PEI:DNA ratio of 3:1 and 4:1). Furthermore, in terms of post-transfection cell viability, the viability of HEK293T cells was significantly higher in the PEI:DNA ratio of 1:1 and 2:1 group in comparison with other PEI:DNA ratios (p value < 0. 001 for PEI:DNA ratio of 3:1 and p value < 0.0001 for PEI:DNA ratio of 4:1). Moreover, GFP expression rate in the transfected HEK293T cells was also detected under a fluorescence microscope. The different PEI:DNA ratios resulted in different GFP expression levels in the transfected cells (data not shown).

### 3.2 Viral titration and transduction optimization

**Figure 2:**
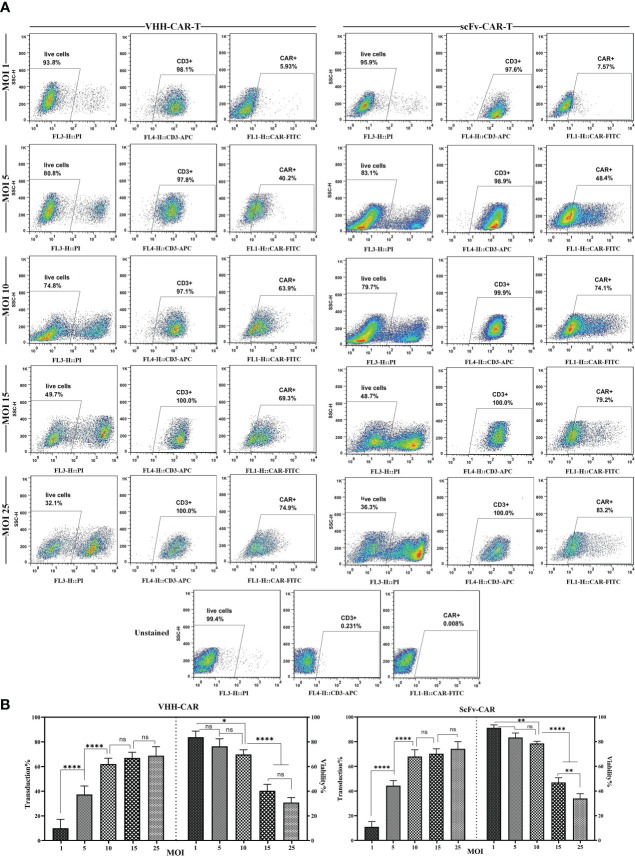
Effects of different MOI on the viability and transduction rate of T cells. **(A)** Flow cytometry plots of the effects of different MOI (1, 5, 10, 15, and 25) on the viability and transduction rate of T cells. **(B)** Statistical analyses of how different MOI result in different viability and transduction rates of T cell in the process of CAR-T production. Data are expressed as the mean ± SD. *p < 0.05, **p < 0.01, and ****p < 0.0001; ns, not significant. Each experiment was carried out in triplicate (n = 3).

In the process of CAR-T manufacturing, it is important to know the concentration of the produced viral particles. In addition, determining the MOI of the produced viral particles helps optimize gene delivery into T cells during CAR-T production. To ensure maximum accuracy and compensate for multiple transduction events per cell, the viral particle titers were calculated by two practical assessments: flow cytometry analysis for the cells that express the integrated transgene and qPCR to assess the relative copy number of the produced lentiviral particles. As the results indicated, there was a close correlation between the expression of GFP, VHH-based CAR molecules, and scFv-based CAR molecules in the corresponding groups and the amount of the produced lentiviral particles as TU/mL (data not shown). MOI of 1, 5, 10, 15, 25 were considered for the transduction of T cells. As the results indicated (**Figure 2**), MOI of 10 mediated the highest rate of transduction alongside the lowest rate of rate of cytotoxicity; therefore, this MOI was selected for the transduction of T cells for the rest of the experiments. In reference to the flow cytometry result analysis, the population of cells were first gated on PI-negative cells (live cells). Next, the PI-negative cells were gated on the expression of CD3 (CD3-positive cells), and then the population of CAR-positive cells was assessed in each cell population.

### 3.3 Lentiviral transduction and assessment of CAR expression rate on the surface of CAR-Ts

**Figure 3:**
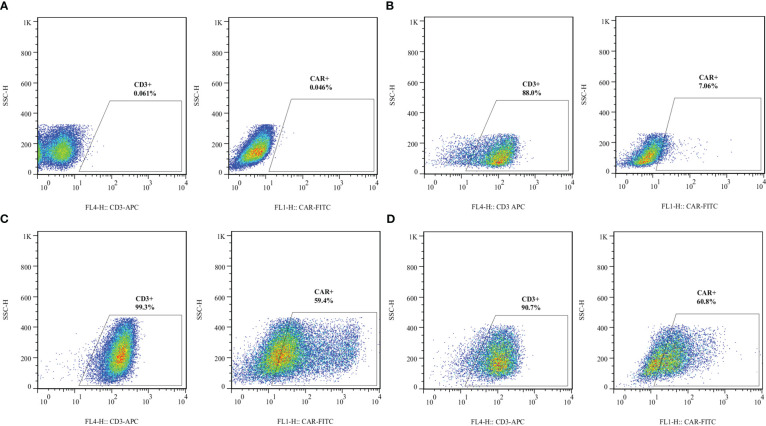
Transduction efficacy of the developed CAR-Ts as assessed by staining surface-expressed CARs in different experimental groups. **(A)** Unstained. **(B)** Non-transduced T cells. **(C)** The expression rate of VHH-based CARs on the surface of VHH-CAR-Ts. **(D)** The expression rate of scFv-based CARs on the surface of scFv-CAR-Ts. Each experiment was carried out in triplicate (n = 3).

Lentiviral transduction of the activated primary T cells resulted in the efficient surface expression of VHH- and scFv-based CARs. In this experiment, we used five different MOI for the lentiviral transduction step. We considered MOI of 1, 5, 10, 15, and 25 for T cell transduction to determine the optimized MOI which could result in the lowest rate of cytotoxicity and highest rate of lentiviral transduction and CAR surface expression. An MOI of 10 was selected as the optimized MOI for the transduction procedures in this study. Flow cytometry was used to evaluate the efficacy of lentiviral transduction by analyzing the surface expression of different CARs. According to the flow cytometry plots presented in **Figure 3**, around 59% of the cells in the VHH-CAR-T group were positive for the expression of VHH-based CARs, whereas about 60% of the cells in the scFv-CAR-T group were positive for the expression of scFv-based CARs.

### 3.4 VHH-CAR-Ts exhibited target antigen-dependent expansion upon co-cultivation with CD19-positive cell lines

**Figure 4:**
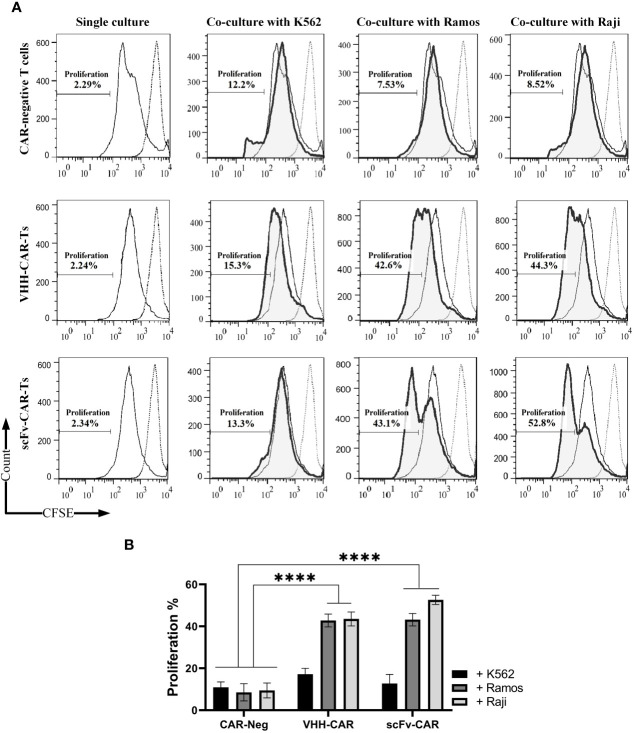
Target antigen-specific activation and expansion of CAR-Ts. **(A)** Flow cytometry assessment of CFSE in CAR-negative T cells, VHH-CAR-Ts, and scFv-CAR-Ts upon their co-cultivation with the CD19-positive target cell lines Ramos and Raji and the CD19-negative cell line K562. **(B)** Statistical analysis between the expansion rate of CAR-negative T cells, VHH-CAR-Ts, and scFv-CAR-Ts upon their co-cultivation with the CD19-positive target cell lines Ramos and Raji and the CD19-negative cell line K562. Data are expressed as the mean ± SD. ****p < 0.0001. Each experiment was carried out in triplicate (n = 3).

Durable disease remission in cancer patients receiving CAR-T therapy is highly dependent on the *in vivo* persistence and expansion of the adoptively transferred CAR-Ts (23). The cytolytic activity or cytokine release of CAR-Ts does not necessarily guarantee that CAR-Ts can have efficient proliferation upon encountering their target cells. Herein, CAR-negative T cells, VHH-CAR-Ts, and scFv-CAR-Ts were labeled with CFSE before co-cultivation with K562, Raji, and Ramos target cells. Of note, the effector cells were not supplemented with IL-2 to be able to assess target antigen-dependent effector cell expansion. 72 hours after co-cultivation, the proportion of CAR-Ts divided was assessed using flow cytometry. According to the results presented in **Figures 4A, B**, VHH-CAR-Ts and scFv-CAR-Ts exhibited a comparable proportion of cells divided over 72 hours of co-cultivation with Raji and Ramos target cells in comparison with the same effector cells co-cultured with K562 cells. Moreover, CAR-negative control cells demonstrated significantly lower expansion rates after 72 hours of co-cultivation with K562, Raji, and Ramos target cells in comparison with VHH-CAR-Ts and scFv-CAR-Ts (p value < 0.0001 for both groups).

### 3.5 VHH-CAR-Ts meditated selective cytolytic reactions against CD19-positive cell line upon co-cultivation

**Figure 5:**
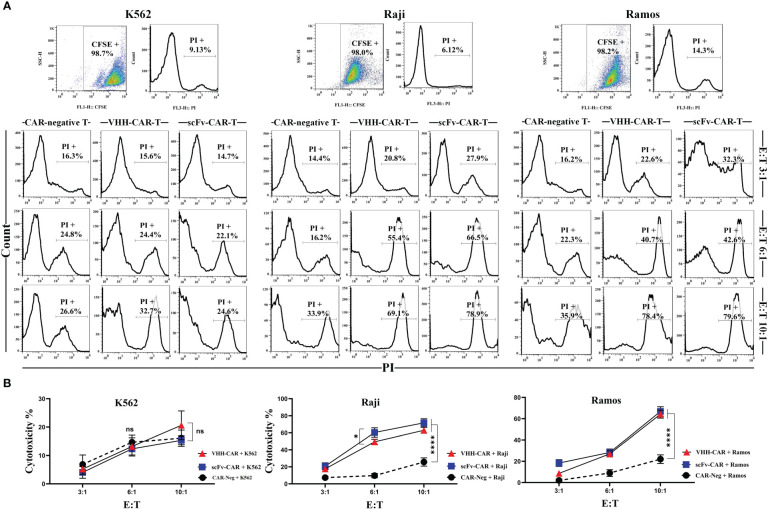
Cytotoxicity of CAR-Ts against the CD19-positive target cell lines Ramos and Raji and the CD19-negative cell line K562. **(A)** Flow cytometry assessment of PI staining in K562, Raji, and Ramos target cells after co-cultivation with CAR-negative T cells, VHH-CAR-Ts, and scFv-CAR-Ts at three different E:T ratios (3:1, 6:1, and 10:1). Each pair of dot-plot and histogram relating to any of the target cell lines at the upper panel of the figure represents their CFSE signal level and viability, respectively, before co-cultivation. **(B)** The linear plot of the cytotoxicity rate of CAR-negative T cells, VHH-CAR-Ts, and scFv-CAR-Ts against the CD19-negative cell line K562 and the CD19-positive target cell lines Raji and Ramos. Data are expressed as the mean ± SD. *p < 0.05 and ****p < 0.0001; ns, not significant. Each experiment was carried out in triplicate (n = 3).

Effector cells (VHH-CAR-Ts and scFv-CAR-Ts, and CAR-negative control T cells) were co-cultured with target cells at three different E:T ratios (3:1, 6:1, and 10:1). Target cells included K562 cells (as CD19-negative cells), and Raji and Ramos cells as (CD19-positive cells). The target-specific cytotoxic activity of the effector cells was assessed after 16 hours of co-cultivation with the target cells. According to the results presented in **Figures 5A, B**, all three types of effector cells demonstrated almost similar rates of cytotoxicity against K562 cells at each of the E:T ratios. In the Raji group, VHH-CAR-Ts mediated a rate of around 20, 55, and 69% damaged target cells at 3:1, 6:1, and 10:1 E:T ratios, respectively. In the same group, scFv-CAR-Ts mediated similar cytotoxicity rates with around 27, 66, and 79% damaged target cells at 3:1, 6:1, and 10:1 E:T ratios, respectively. These number were at least two-fold higher than what was observed in the CAR-negative control cells group except for VHH-CAR-Ts at 3:1 E:T ratio.

In the Ramos group, all three types of effector cells demonstrated cytotoxic behavior similar to the Raji group. In detail, VHH-CAR-Ts mediated a rate of around 22, 40, and 78% damaged target cells at 3:1, 6:1, and 10:1 E:T ratios, respectively. Furthermore, scFv-CAR-Ts mediated similar cytotoxicity rates with around 32, 42, and 79% damaged target cells at 3:1, 6:1, and 10:1 E:T ratios, respectively. Similar to the Raji group, these numbers were almost two-fold higher than what was observed in the CAR-negative control cells group except for VHH-CAR-Ts at 3:1 E:T ratio. Overall, it can be stated that scFv-CAR-Ts mediated slightly stronger target-specific cytotoxicity than VHH-CAR-Ts against Raji and Ramos target cells. Of note, it was also discovered that efficient antitumor activity of CAR-Ts could be achieved at higher E:T ratios.

### 3.6 VHH-CAR-Ts secreted almost similar levels of IFN-γ, IL-2, and TNF-α upon encountering CD19-positive cell lines as their FMC63-based counterparts

**Figure 6:**
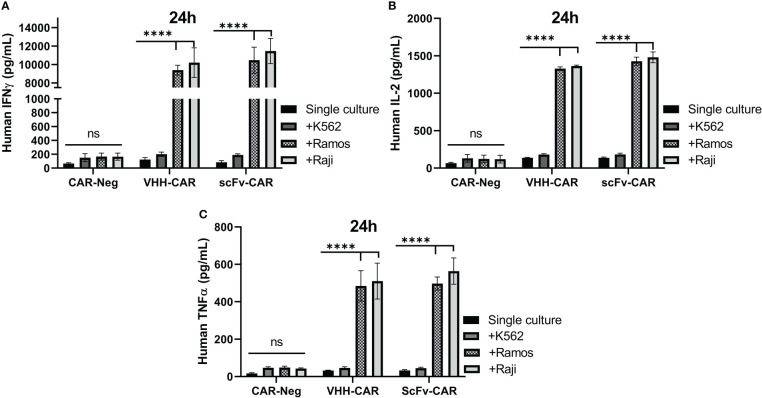
The cytokine production and secretion of CAR-negative T cells, VHH-CAR-Ts, and scFv-CAR-Ts upon their co-cultivation with the CD19+ target cell lines Ramos and Raji and the CD19- cell line K562 at an E:T ratio of 1:1. **(A)** Human INF-γ. **(B)** Human IL-2. **(C)** Human TNF-α. Data are expressed as the mean ± SD. ****p < 0.0001; ns, not significant. Each experiment was carried out in triplicate (n = 3).

Target-antigen dependent cytokine production and secretion of VHH-CAR-Ts, scFv-CAR-Ts, and CAR-negative control cells were measured using ELISA after 24 hours of co-cultivation with K562, Raji, and Ramos target cells at 6:1 E:T ratio. As presented in **Figure 6**, separate analyses showed that both scFv-CAR-Ts and VHH-CAR-Ts secreted higher levels of human IFN-γ, human IL-2, and human TNF-α in comparison with CAR-negative T cells upon co-cultivation with the CD19-positive Raji and Ramos cells but not upon co-cultivation with the CD19-negative K562 cells or during single culture of the effector cells. The secretion of human IFN-γ, human IL-2, and human TNF-α by VHH-CAR-Ts upon co-cultivation with Raji and Ramos cells were significantly higher than their levels upon co-cultivation with K562 cells or during single culture of the VHH-CAR-Ts as negative control (p value < 0.0001, for all of the groups). The same pattern was observed in regards to the secretion of IFN-γ, IL-2, and TNF-α by scFv-CAR-Ts upon co-cultivation with Raji, Ramos, and K562 cells or during their single culture as negative control (p value < 0.0001, for all of the groups). Moreover, VHH-CAR-Ts and scFv-CAR-Ts secreted almost similar levels of IFN-γ, IL-2, and TNF-α while co-cultured with the CD19-negative cell line K562 and the CD19-positive cell lines Raji and Ramos. This could accentuate the fact that VHH-CAR-Ts can be as potent as scFv-CAR-Ts in terms of secreting certain proinflammatory cytokines in response to the presence of CD19-positive cell lines.

## 4 Discussion

CAR-T therapy has been considered a revolution in the treatment of patients with certain hematologic malignancies. B-ALL, DLBCL, FL, MCL, and MM patients are now the beneficiaries of this cancer treatment modality that is itself a result of intricate protein engineering and cancer immunotherapy. Since 2017 that the first CAR-T product was granted approval for medical use by the US FDA, numerous clinical trials have started assessing the antitumor efficacy and safety of this treatment method for patient with different oncological and immunological indications. Among those CAR-T therapy clinical trials that aim to investigate such products for the treatment of patients with blood-based cancer, CD19, CD20, CD22, and BCMA are the most frequent target antigens. In this study, we constructed a CD19-redirected CAR whose targeting domain was based on a VHH. Currently, most of the CAR-Ts being assessed in clinical trials harbor scFvs as their targeting domains. The light chain (V_L_) and heavy chain (V_H_) of an scFv are fused together by the means of a flexible linker. Due to the fact that scFvs are recombinant fragments derived from full-length mAbs, there have been reports of their inherent tendency to aggregate on the surface of CAR-Ts (24–26). This occurrence, termed “*tonic signaling*”, leads to the antigen-independent activation and downstream singling of CAR-Ts, resulting in their exhaustion (24–26). For instance, Landoni et al. conducted an experiment to modify the amino acid sequences of the frameworks regions of CAR targeting domains to overcome the issue of CAR tonic signaling (26). Tonic signaling debilitates the antitumor efficacy of the infused CAR-T cells, leaving room for disease progression (23). As a solution, single-domain antibodies could be applied as the antigen-recognition domain of CAR constructs, since there have not been any reports regarding the tonic signaling of VHH-based CAR-Ts. However, broader investigations are warranted in this regard to clearly assess whether VHH-based CAR constructs tend to aggregate on the surface of CAR-Ts to mediate tonic signaling. This topic could be a direction for future research.

Most of the antigen-recognition fragments of CAR-Ts are derived from animal-origin antibodies which might be considered immunogenic while administered to human subjects (23, 27, 28). Several investigations have reported anaphylaxis in patients receiving CAR-T products whose targeting domains were based on murine scFvs (27, 28). Such reactions result in the production of neutralizing antibodies in the body of the recipients, impairing the antitumor efficacy of the infused CAR-Ts and the consequent abrogation of all CAR-T-related therapeutic benefit as a result of their elimination from circulation (23, 27, 28). To overcome this issue, scientists proposed the strategy of humanizing such CAR targeting domains for the generation of humanized CAR-Ts or using targeting domains derived from fully-human mAbs (23, 29–32). To date, several humanized CAR-Ts are being assessed for their safety and antitumor potential in various clinical trials (NCT02782351, ChiCTR1800017401, etc.) (18, 23, 29–31). Since the antigen-recognition domain of our VHH-CAR-Ts was derived from a camelid heavy-chain-only antibody, the issue of immunogenicity might be one of the limitations of this study which warrants further investigations.

Aside from the CD19 antigen, VHH-based CAR-Ts could also be used for the simultaneous targeting of other hematologic malignancy-associated target antigens such as CD20, CD22, CD123, CD30, etc (17, 20). Of note, two VHHs could be fused and engineered into the construct of any given CAR to enable CAR-Ts to target two different antigens or two different epitopes of a single antigen. In particular, our laboratory experience demonstrated that oligoclonal CAR-Ts, equipped with oligoclonal anti-HER2 nanobodies, exhibit higher proliferation, cytokine secretion, and tumoricidal capacity in comparison with CAR-Ts harboring each of the single nanobodies that constitute the oligoclonal CAR (33). To date, several clinical investigations are assessing the safety and antitumor efficacy of CD19/CD20- or CD19/CD22-redirected CAR-Ts for the treatment of individuals with blood-based cancers (NCT03233854, NCT03019055, and NCT03196830). The importance of such applicability is accentuated in the case of CD19 downregulation or loss as reported by various recent investigations (34–36). As accumulating evidence suggests, malignant cells undertake intricate mechanisms to escape immune surveillance (34–36). Such mechanisms entail alternative splicing of the target antigen (such as CD19) recognized by CAR-Ts in a way that the epitope recognized by the targeting domain is no longer present in the target antigen (35, 36). In such cases, a CAR-T product equipped with a different targeting domain whose epitope is still present in the alternatively-spliced target antigen should be taken into consideration (34). This occurrence further highlights the importance of having different CD19-redirected CAR-T products, so that patients with CD19-positive malignancies can still continue to benefit from this treatment modality as long as they have not experienced CD19 loss. In this regard, a pilot study was conducted (NCT02975687) to assess the safety and feasibility of CAR-Ts that target a different CD19 epitope, rather than that recognized by FMC63, in pediatric and adult R/R B-ALL patients (34). The results demonstrated that 18 out of 20 patients (90%) achieved complete remission (CR)/incomplete count recovery (CRi) within 28 days, thus highlighting the fact that the antileukemic potential of these CAR-Ts can be as satisfactory as those of conventional CD19-redirected CAR-T products (34).

scFv development requires the use of synthetic linker peptides for fusing V_H_ and V_L_ domains (37). The presence of such linkers in the construct of scFvs can be immunogenic and may trigger host immunological defensive reactions through the production of neutralizing antibodies (35, 38). In the case of nanobodies, the absence of these synthetic linker peptides leaves no room for the mediation of host immune responses. Similarly, VHH-based CAR-Ts have been investigated in various clinical stages and there have not been any reports regarding their immunogenicity and post-infusion production of neutralizing antibodies against them. Also, limited studies have indicated that nanobodies might be immunogenic in a negligible way when used as the targeting domain of CAR-Ts redirected against HER2 (39). It is worth mentioning that some researchers have pointed out the fact that nanobodies are more well-tolerated and less immunogenic for in-human use since their sequences are highly similar to those of human V_H_ (40). Another structural difference between scFvs and VHHs is the long CDR3 of nanobodies enabling them to bind specific epitopes that are not accessible to human or mouse mAbs, or scFvs (41–43). Additionally, the humanization process of nanobodies is considered to be much simpler than that of scFvs due to the fewer amino acid residue substitutions required for the humanization of nanobodies (44). Such fewer number amino acid residue substitutions can reduce the risk of affinity loss after humanization (18, 23). Also, unlike nanobodies, the large DNA fragments of scFvs can adversely affect the efficiency of transfection and viral packaging in the process of CAR-T generation (45–47).

Moreover, studies have also reported mutations in the genes encoding the target antigens which lead to antigen loss, to overcome which CAR-Ts should be programmed to target different antigen(s) (35). In addition to antigen loss, Ruella et al. have reported a rare incidence in the manufacturing process of CD19-redirected CAR-Ts which rendered a patient resistant to CD19-based CAR-T products, despite the consistent expression of this antigen (48). In detail, a single leukemic cell was accidentally transduced in the process of CAR-T manufacturing which resulted in the expression of CAR molecules on its surface, and their consequent engagement with CD19 on the surface of the malignant cell (48). Following administration into the patient, this single leukemic cell expanded and resulted in a vast population of CD19-positive leukemic cells resistant to CD19-redirected CAR-T products (48). In such cases, different target antigens should be taken into consideration. According to the findings of a Phase I dose-escalation study (NCT04088890), three patients with large B-cell lymphoma achieved CR following treatment with CD22-redirected CAR-Ts after they experienced relapse from their previous treatment with CD19-redirected CAR-Ts (49).

One of the benefits of developing potent CD19-redirected CAR-T products is that they can be applied for the eradication of a wide range of hematologic malignancies owing to the constitutive expression of CD19. For instance, Novartis’ *Kymriah* has been granted permission by the US FDA as a third-line treatment for B-ALL, DLBCL, and FL. Aside from the mentioned oncological indications, CD19-redirected CAR-T products are currently being assessed against various CD19-positive hematologic cancers. As an example, Juno Therapeutics’ *JCAR017* is currently being evaluated in an open-label Phase I/II clinical trial (NCT03331198) for the treatment of 259 individuals with R/R chronic lymphocytic leukemia (CLL) or small lymphocytic lymphoma (SLL). According to a study by Xue et al., a patient with R/R classical Hodgkin’s lymphoma (cHL) was treated with low doses and repeated infusions of CD19- and CD30-redirected CAR-Ts which resulted in the prolonged progression-free survival (PFS) of the patients without any severe treatment-related toxicities (50). Xue and colleagues further highlighted the importance of combination CAR-T therapy for the treatment of cHL (50). CD19 might also be considered a suitable target antigen in particular subtypes of MM cells, despite being absent from the dominant MM cells (51). According to a clinical study by Garfall and colleagues (NCT02135406), 2 out of 10 patients (20%) with R/R MM underwent CD19-redirected CAR-T therapy (CLT019) following autologous stem cell transplantation (ASCT) and melphalan-based salvage therapy, and experienced remarkably more prolonged PFS than when they only underwent ASCT as prior therapy (479 versus 181 days for one patient, and 249 versus 127 days for the other, respectively) (51). Based on the findings of a clinical investigation by Zhou et al., CD19 might also be considered as a suitable target antigen even for the treatment of patients with Burkitt’s lymphoma (52). In detail, 3 out of 6 patients with Burkitt’s lymphoma, who underwent CD19- and CD22-redirected CAR-T therapy, achieved an objective response (two partial responses; one CR) (52). Clinical investigations, such as those exemplified here, accentuate the suitability of CD19 as a target antigen in a wide range of blood-based cancers alongside the applicability of CD19-redirected CAR-T products as possible treatment options.

VHH-based CAR-Ts were first introduced as alternatives to overcome the limitations of scFv-based CAR-Ts. In the early days, concerns were raised regarding the effectiveness of VHH-based CAR-Ts (53). So far, numerous studies have demonstrated that VHH-based CAR-Ts are capable of mediating specific target antigen-dependent cytotoxicity against various types of malignancies both in preclinical and clinical investigations (53, 54). In detail, the antitumor activity and efficient effector function of VHH-based CAR-Ts have been highlighted in both hematologic malignancies and solid tumors (20). To our knowledge, this is the first study comparing the antitumor activity of VHH-based CD19-redirected CAR-Ts with their FMC63-based counterparts. To date, other hematologic malignancy target antigens such as CD20, CD7, CD33, CD38, and BCMA have also been targeted using VHH-based CAR-Ts, and it has been demonstrated that these engineered effector cells can mediate target antigen-dependent antitumor activity and efficient cytokine secretion, and they have also been proven to be well-tolerated and capable of mediating satisfactory CR rates in clinical studies (53–59). Our *in vitro* results of the antitumor activity of VHH-CAR-Ts are consistent with these clinical trials even though some of the mentioned studies target non-B cell-specific target antigens (20). It is also worth mentioning that *ciltacabtagene autoleucel* (also known as *cilta-cel* or *CARVYKTI*) is a CAR-T product based on two single-domain antibodies which was approved on February 28, 2022 by the US FDA for the treatment of certain adult patients with R/R MM [12]. This was the first single-domain antibody-based CAR-T product approved for medical use in the US. Though this product targets BCMA, such favorable clinical approvals can highlight the potential of single-domain antibody-based CAR-Ts, at least in hematologic malignancies.

## 5 Conclusion

CAR-T therapy has proven to be a highly effective treatment modality for certain patients with particular blood cancers who have failed to respond to prior conventional therapies. To date, four CD19-redirected CAR-T products have been granted the US FDA approval for the treatment of certain patients, which highlights the potential applicability of this cancer treatment modality and the suitability of CD19 as a target antigen for the treatment of CD19-positive malignancies. In this study, we developed and characterized VHH-based CD19-redirected CAR-Ts, and demonstrated that these engineered effector cells could be as potent as their FMC63 scFv-based counterparts *in vitro*. Future studies will be focused on assessing the safety and antitumor efficacy of our VHH-CAR-Ts in xenograft animal models to further validate the findings of this investigation.

## Data availability statement

The original contributions presented in the study are included in the article/supplementary material. Further inquiries can be directed to the corresponding author.

## Ethics statement

The studies involving human participants were reviewed and approved by Tarbiat Modares University Research Ethics Committee. The patients/participants provided their written informed consent to participate in this study.

## Author contributions

FN: Data curation, Formal analysis, Investigation, Methodology, Project administration, Validation, Visualization, Writing - original draft, Writing - Review and Editing. PSK: Data curation, Formal analysis, Investigation, Methodology, Validation, Visualization, Writing - original draft, Writing - Review and Editing. FR: Conceptualization, Data curation, Formal analysis, Funding acquisition, Methodology, Project administration, Resources, Software, Supervision, Validation. All authors contributed to the article and approved the submitted version.
